# From Bacteria to Cancer, the Dedifferentiation Hypothesis of Mitochondria to Immortal Multipotent Cancer Stem Cells

**DOI:** 10.3390/bioengineering13101139

**Published:** 2026-09-29

**Authors:** Ciro Gargiulo Isacco, Van Hung Pham, Kieu C. D. Nguyen, Toai Cong Tran, Emilio Jirillo, Luigi Santacroce

**Affiliations:** 1 Section of Microbiology and Virology, Department of Interdisciplinary Medicine, School of Medicine, University of Bari “Aldo Moro”, 70124 Bari, Italy; drkieukaren@gmail.com (K.C.D.N.); emilio.jirillo@uniba.it (E.J.); luigi.santacroce@uniba.it (L.S.); 2 Viet Nam Research and Development Institute of Clinical Microbiology, Ho Chi Minh City 756000, Vietnam; phhvan.nkbiotek@gmail.com; 3 Basic Medical Sciences Faculty, Pham Ngoc Thach University of Medicine, Ho Chi Minh City 740500, Vietnam; trancongtoai@pnt.edu.vn

**Keywords:** mitochondria, cancer, differentiation, mtDNA, nDNA, mtRNA, oxidative phosphorylation (OXPHOS), NUMTs, ROS, retrotransposons, transposons, Telomere-Telomerase, TERT, TEs, L1, TPP1, TIN2

## Abstract

**Background**: Carcinogenesis has traditionally been attributed to the accumulation of somatic mutations in nuclear genes regulating cell proliferation, survival, and apoptosis. Although hereditary genetic predispositions account for less than 10% of cases, the extensive heterogeneity of the somatic mechanisms driving tumorigenesis—including subsets of neoplasms lacking canonical nuclear mutations—suggests that non-mutational mechanisms and organelle dynamics significantly influence tumor onset. Emerging evidence increasingly supports the concept that mitochondrial dysfunction and metabolic reprogramming are essential factors in tumor development, rather than merely downstream consequences of nuclear genetic alterations. While current literature establishes mitochondrial alterations as crucial co-initiators and facilitators of early transformation, defining whether metabolic failures may eventually act as primary trigger remains an ongoing area of investigation. Within this perspective, the “Endosymbiotic/Evolutionary Theory” provides a useful heuristic model, since it conceptualizes metabolic rewiring not as a literal genetic reversal, but as an adaptive shift toward ancestral, highly glycolytic survival programs when oxidative microenvironments are compromised. **Methods**: We conducted a comprehensive theoretical and comparative analysis of mitochondrial biology, interrogating the structural, functional, and evolutionary parallels between mitochondria, intracellular bacteria, and viruses within the conceptual context of the endosymbiotic theory. Particular attention was given to mitochondrial proteins associated with organellogenesis, bioenergetic control, and molecular mechanisms facilitating mitochondrial–nuclear communication, including the integration of mitochondrial DNA (mtDNA) into nuclear DNA (nDNA) as nuclear mitochondrial DNA segments (NUMTs). **Results**: Under sustained bioenergetic stress, mitochondrial retrograde signaling can achieve regulatory dominance over the nucleus, driving a functional dissociation between organellar metabolic state and nuclear control. While not autonomous in a strict sense, this shift echoes the evolutionary legacy described by the endosymbiotic theory, whereby mitochondria act as semi-independent bioenergetic systems capable of imposing profound effects on nuclear gene expression and cellular fate. The resulting state is characterized by metabolic reconfiguration, loss of differentiation fidelity, and the acquisition of cancer stem cell–like properties. This process may unfold through a sequence of interrelated events: (1) persistent endogenous or exogenous stressors that challenge cellular homeostasis; (2) establishment of a chronic inflammatory state and sustained redox imbalance; (3) progressive disruption of the cellular microenvironment and inter-organelle communication; (4) emergence of a permissive pre-neoplastic niche characterized by impaired oxidative phosphorylation (OXPHOS), altered mitochondrial dynamics, and sustained retrograde signaling; (5) activation of adaptive survival pathways and escape from canonical cell death and checkpoint controls; and (6) progression toward a dedifferentiated, stem-like cellular phenotype associated with metabolic plasticity and increased resilience. **Conclusions**: We recommend that cancer be reframed as a dedifferentiated cellular state primarily driven by mitochondria-derived signaling, reflecting the organelle’s ancestral autonomy and the reactivation of evolutionarily conserved mechanisms involving transposons, retrotransposons, Long Interspersed Nuclear Element-1 (L1), transposable elements (TEs), reactive oxygen species (ROS), and mtDNA–nDNA crosstalk, including the generation of nuclear mitochondrial DNA segments (NUMTs). This perspective extends the canonical view of predominantly nuclear-driven oncogenesis by reframing carcinogenesis as a bioenergetic and bio-evolutionary process, thereby highlighting mtDNA–nDNA interactions and mitochondrial signaling as central targets for cancer prevention, diagnosis, and therapy.

## 1. Introduction

According to prevailing oncology models, cancer arises from complex, multifactorial aberrations involving both external and internal factors. These include chronic inflammation, infections, genetic mutations, and random somatic changes affecting proto-oncogenes, tumor suppressor genes, and inherited genetic defects [1,2,3]. Although numerous mutations have been identified across different tumor types, so-called *driver gene* mutations are generally regarded as the principal cause of cancer. Based on this assumption, tumor cells are also thought to be dependent on the presence of inherited genetic variations [2,3,4,5].

However, this paradigm presents several limitations, as it does not fully account for the biological complexity and phenotypic heterogeneity of cancer. For example, the oxidative phosphorylation (OXPHOS) system, which is increasingly recognized as a central regulator of carcinogenesis, consists of hetero-oligomeric complexes (I, III, IV, and V) whose subunits are encoded by both the nuclear and mitochondrial genomes [6,7]. This dual genetic control highlights the importance of mitochondrial–nuclear crosstalk in maintaining cellular homeostasis and suggests that malignant transformation cannot be explained solely by alterations in the nuclear genome [6,7]. Furthermore, clonal expansion of canonical driver mutations has been documented in histologically normal human tissues, whereas a subset of tumors lacks identifiable alterations in established driver genes or obvious chromosomal abnormalities. For example, analyses of Br20P glioblastoma tissue samples failed to detect mutations in *TP53*, *PIK3CA*, or *RB1* [6,7,8]. Although additional pathogenic variants may reside within non-coding regulatory regions of the genome, the absence of classical driver mutations in certain cancers raises important questions regarding the relative contribution of nuclear genetic alterations alone to the initiation and maintenance of the malignant phenotype [8,9].

These observations suggest that greater attention should be directed toward cellular components that are integral part of eukaryotic cells while retaining a degree of functional and genetic autonomy, particularly mitochondria [10]. Such a conceptual shift is supported by the endosymbiotic theory, according to which modern eukaryotic cells arose through a mutually beneficial cooperation between an ancestral archaeal host and an α-proteobacterial endosymbiont that ultimately evolved into the mitochondrion [10]. This evolutionary origin is supported by the structural, biochemical, and genetic similarities shared between mitochondria and intracellular pathogens, including their circular genome, bacterial-type ribosomes, and replication machinery, as well as their evolutionary relationship with chloroplasts [10,11]. Additional support comes from the observation that several antimicrobial agents, including selected antibiotics, antivirals, and antifungals, can directly or indirectly modulate mitochondrial function by targeting evolutionarily conserved metabolic pathways or organellar processes. In different types of malignancies, some of these agents have also demonstrated antitumor activity [10,11].

Accordingly, we propose that the central question in cancer biology should shift from “*How does cancer grow and metastasize?*” to “*What is a cancer cell, and which biological processes are involved in its rise?*” We hypothesize that carcinogenesis is initiated through a progressive sequence of mitochondrial reorganization and metabolic adaptations that precede overt malignant transformation. These changes enable metabolically stressed cells to overcome long-term homeostatic constraints, undergo cellular reprogramming, and progressively acquire the hallmark properties of cancer stem cells, including multipotency.

## 2. Rethinking Cancer Through a Metabolic Lens

The mitochondrial metabolic trait of cancer proposed by Seyfried and colleagues tend to open a new scenario in modern oncology [1,2]. Seyfried’s model proposes that impaired mitochondrial OXPHOS and altered energy metabolism constitute early events in tumorigenesis, preceding nuclear genomic instability and aberrations. Mitochondrial dysfunction creates a metabolic transition in which cells progressively increase their dependence on substrate-level phosphorylation and fermentative metabolism, including glycolysis and glutaminolysis [3,4]. Rather than representing merely cellular damage, this metabolic adaptation may provide the energetic and biosynthetic flexibility required for cellular reprogramming, survival under metabolic stress, and the subsequent acquisition of cancer stem cell properties. The impaired metabolism increases reactive oxygen species (ROS), which cause the genomic instability (mutations) often seen in cancer cells, rather than the mutations that cause cancer [5,6].

Of note, glycolysis and glutaminolysis, in normal condition are managed by either nucleus or mitochondria. While glycolysis takes place in the cytosol and is transcriptionally regulated by nuclear factors, including hypoxia-inducible factor-1 (HIF-1) and c-Myc, these transcriptional programs are increasingly recognized as operative mechanisms under mitochondrial regulatory influence through retrograde signaling and metabolic feedback [5,6]. Glutamine supports the tricarboxylic acid (TCA) cycle in the mitochondria, directly relating it to mitochondrial health and metabolic adaptation. Glutaminolysis is primarily a mitochondrial process, regulated by the nucleus (via Myc/miR-23a/b), it increases when mitochondrial function is impaired or in consequence of higher energy request, such as in cancer [5,6].

Therefore, under conditions of nutrient starvation or hypoxia, mitochondria function as metabolic sensors that transmit retrograde signals to the nucleus, triggering HIF-1- and c-Myc-dependent transcriptional reprogramming and a temporary shift from OXPHOS to glycolysis, keeping stable cellular homeostasis during metabolic stress [5,6].

## 3. From Endosymbiosis to Coevolution: The Mitochondria–Microorganisms Affinity

To understand the fundamental role of mitochondria in cellular metabolism and immune responses, it is essential to consider their evolutionary origin and intrinsic similarities to microorganisms. Mitochondria retain several distinctive features consistent with their bacterial ancestry and are widely regarded as descendants of an ancestral α-proteobacterium acquired by an early eukaryotic cell through endosymbiosis. The progressive oxygenation of Earth’s atmosphere created new ecological and metabolic opportunities, facilitating biological diversification and the emergence and expansion of oxygen-dependent respiration across diverse lineages, including microorganisms [12].

Mitochondria are dynamic, often elongated or ellipsoidal organelles, typically ranging from approximately 0.5–10 μm, and possess their own small, circular DNA genome. Although the vast majority of mitochondrial proteins are now encoded by nuclear genes following extensive endosymbiotic gene transfer, mitochondrial genomes have retained a limited set of genes essential for oxidative phosphorylation and organelle function [13,14]. Mitochondrial DNA replicates within the organelle and is transmitted through mitochondrial division, or fission, during which pre-existing mitochondria give rise to daughter organelles. This mode of propagation, together with the circular organization of mitochondrial DNA and its bacterial-like replication machinery, represents one of the most compelling molecular signatures of mitochondrial ancestry [15,16,17].

**Figure 1:**
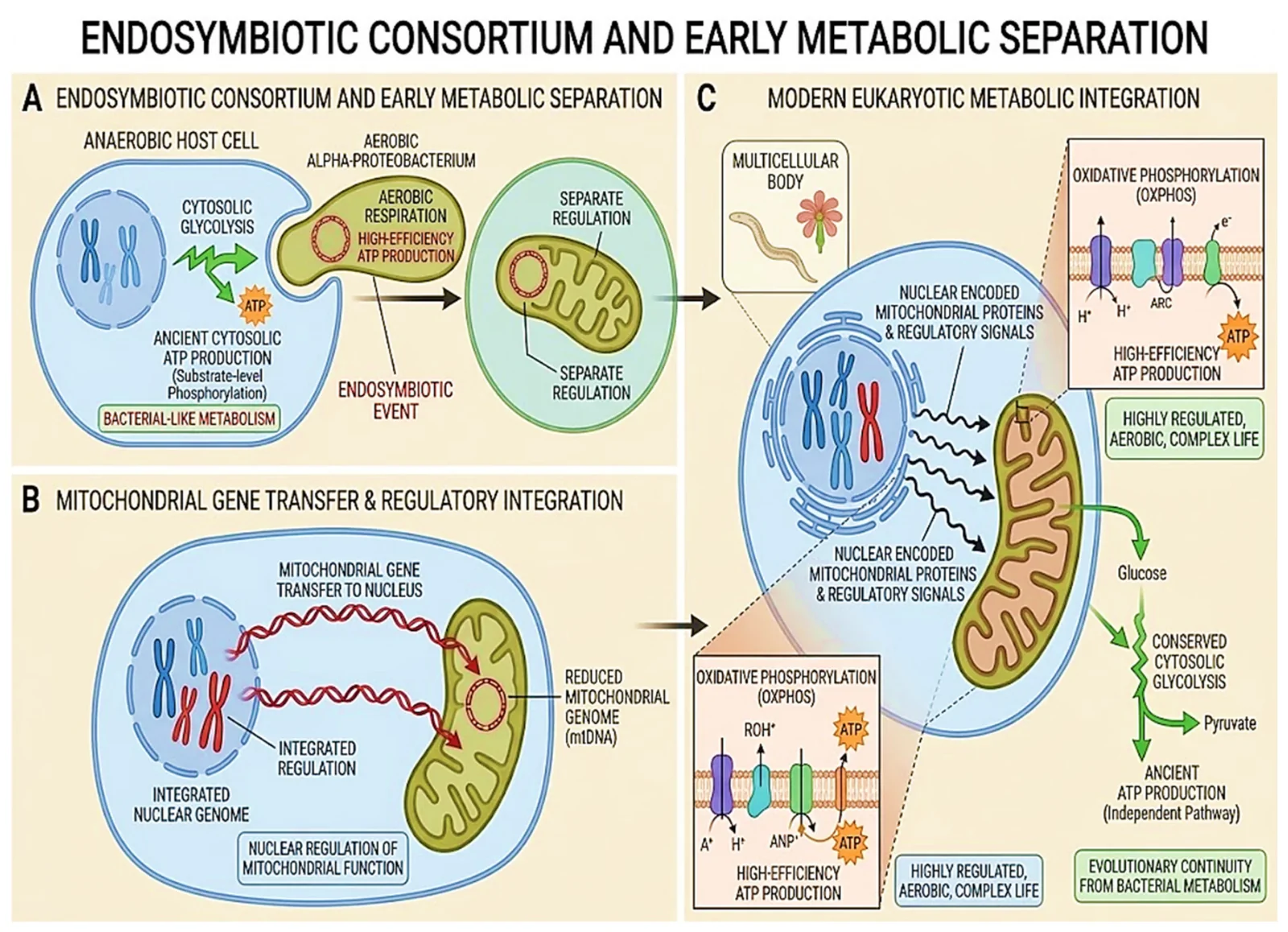
Panel (**A**): In the initial endosymbiotic event, we highlight the early separation between the aerobic alpha-proteobacterium (the future mitochondrion) and the anaerobic host cell, which relies exclusively on cytosolic glycolysis. (**B**): In evolutionary gene transfer, we visualize the transition of most mitochondrial genes to the nuclear genome, establishing the nucleus as the master regulatory center while maintaining a reduced mitochondrial genome (mtDNA) that preserves a certain degree of cellular autonomy. (**C**): Modern integrated metabolism, comparing the ancient anaerobic glycolytic pathway (conserved from the original bacterial metabolism) with the complex aerobic oxidative phosphorylation (OXPHOS), now under dual nuclear and mitochondrial control. This integration ultimately enables the highly efficient energy production that supports multicellular complexity (Gargiulo-Isacco Ciro).

The subsequent transfer of mitochondrial genes to the nuclear genome represented a pivotal evolutionary transition, integrating mitochondrial function with nuclear regulation and enabling the emergence of increasingly complex eukaryotic and multicellular life [17,18]. This evolutionary integration coupled cellular survival and proliferation to mitochondrial respiratory efficiency, while preserving glycolysis as an ancient and largely autonomous cytosolic energy-generating pathway. These deeply conserved metabolic programs sustain ATP production through cytosolic substrate-level phosphorylation, underscoring the evolutionary continuity between bacterial and eukaryotic energy metabolism (Figure 1) [17,18].

Mitochondrial genomes are shorter than those of their prokaryotic ancestors, and part of their translation machinery shares several protein components with the nucleus [12,13]. Consistent with the endosymbiotic theory, the ancestral α-proteobacterial endosymbiont is thought to have harbored a fully functional OXPHOS system prior to endosymbiosis. The core components and bioenergetic principles of this respiratory machinery remain highly conserved in modern mitochondria, underscoring their bacterial ancestry [13]. Despite mtDNA encoding core ancestral components of the electron transport chain, the majority of OXPHOS complexes are chimeric assemblies of mitochondrial and nuclear gene products, that emphasizes a deep coevolutionary and functional integration of mitochondrial–nuclear systems within eukaryotic cells [13,14].

In addition, modern mitochondria maintained retrograde signaling to alert the nucleus to functional stress, prompting adaptive gene expression tailored to microenvironmental changes and adverse stimuli [15,16,17,18]. A core set of mitochondrially encoded proteins continues to populate the organelle’s membranes, intermembrane space, and matrix [19]. Beyond energy production, mitochondria regulate nuclear gene expression by supplying key metabolites, such as acetyl-CoA and *α*-ketoglutarate, that serve as substrates and cofactors for histone methylation, phosphorylation, and acetylation [20,21]. This dynamic interplay between metabolic reprogramming and epigenetic remodeling represents a fundamental principle underlying the evolution of life, driving cellular adaptation and phenotypic plasticity and enabling organisms to respond to perduring environmental challenges [22,23].

The entire maintenance of the eukaryotic genome relies on mitochondrial function to meet cellular energy demands [23,24]. Mitochondria generate ATP through OXPHOS, supplying the energy required for numerous cellular processes and activities, including nucleocytoplasmic transport and the maintenance of protein synthesis [23,24]. When OXPHOS is inhibited, ATP depletion disrupts nuclear import–export mechanisms, thereby impairing nuclear homeostasis [24,25]. Although cellular metabolism shifts toward glycolysis under hypoxia or mitochondrial respiratory dysfunction, glycolytic ATP remains indispensable for mitochondrial preservation. Cytosol-derived ATP can be transported into mitochondria via adenine nucleotide translocase 2 (ANT2) and consumed by the reverse operation of the F_1_F_0_-ATP synthase to sustain the mitochondrial membrane potential (ΔΨm). Thus, glycolysis may serve as an adaptive metabolic response that reduces dependence on mitochondrial OXPHOS, allowing cells to maintain energy homeostasis during mitochondrial stress until respiratory activity is restored or alternative adaptive programs are established [24,25,26].

### 3.1. Structural Determinants of Mitochondrial Mobility and Heterogeneity

The hybrid nature of mitochondria, heritage from the evolutionary integration of an ancestral *α*-proteobacterial endosymbiont with early eukaryotic host cells, confers a degree of metabolic plasticity evocative of modern microorganisms. Across human cells, the mitochondrial network exhibits substantial ultrastructural heterogeneity, characterized by adjustments in mitochondrial abundance, cristae architecture, membrane lipid composition, electron transport chain (ETC) activity, and mitochondria-associated membranes (MAMs) [27,28,29]. MAMs are of particular importance, since they are specialized contact sites between the endoplasmic reticulum (ER) and mitochondria that coordinate several essential cellular processes, including calcium transfer, phospholipid exchange, and the trafficking of metabolic intermediates and nucleotides [28,29]. Although MAMs represent a distinctive eukaryotic innovation, several proteins and molecular machineries associated with mitochondrial contact sites retain deeply conserved structural and functional features that reflect the evolutionary history of mitochondria. By coordinating mitochondrial metabolism, membrane remodeling, fission and fusion, autophagy, and apoptotic signaling, MAMs enable mitochondria to rapidly adapt their structure and function to changes in nutrient availability, energy demand, oxidative stress, and cellular signaling [29,30,31,32]. Persistent MAM remodeling may contribute to an increase in mitochondrial ROS production, altered calcium signaling, and impaired OXPHOS, which in turn can activate adaptive pathways that favor a shift from mitochondrial oxidative metabolism toward substrate-level ATP production, predominantly through glycolysis and glutaminolysis [29,30,31,32].

### 3.2. Nucleus-Mitochondria Cellular Energy, Evolutionary Legacy, and Epigenetic Remodeling Towards Cancer

Cellular energy production shifts predominantly toward cytosolic substrate-level phosphorylation, while mitochondrial activity is maintained through metabolite exchange and adaptive rewiring of the TCA cycle [33,34,35,36]. Their capacity to transfer and integrate mtDNA fragments into the nuclear genome may contribute to structural genomic changes by altering the regulation of tumor suppressor genes, facilitating oncogene activation, and ultimately promoting tumorigenesis [37,38]. Consistently, analyses of human cancer genomes have demonstrated that chromosomal rearrangements are frequently associated with the somatic insertion of mtDNA fragments into nuclear DNA [37,38,39,40,41].

Nevertheless, spatial organization supports this functional interplay between nucleus and mitochondria. Mitochondria are often localized in close proximity to the nucleus, forming perinuclear clusters that can be observed across multiple cell types by electron microscopy [42,43]. Although physical barriers generally limit direct contact, the outer mitochondrial membrane may come into proximity with the nuclear envelope. This spatial arrangement enables rapid and efficient retrograde signaling, easing adaptive responses that include mitogenesis, mitophagy, and potentially nucleophagy [44,45]. Such interactions have been reported in leukemic cells, suggesting that transient structural coupling may occur under pathological conditions [43,44].

Another important aspect of mitochondria–nucleus communication involves specific transcriptional mediators, including nuclear respiratory factors 1 and 2 (NRF1 and NRF2), which coordinate nuclear gene expression in line with mitochondrial activity. Both NRF1 and NRF2 are transcription factors, encoded in the nucleus but responsive to mitochondrial signals, regulating the expression of genes essential for mitochondrial biogenesis, respiratory function, and mitochondrial DNA maintenance [45,46]. He and colleagues proposed that, beyond their established roles in cellular stress responses and mitochondrial regulation, NRF1 and NRF2 are increasingly recognized as critical regulators of cancer cell metabolism and progression [45,46,47]. NRF2 promotes metabolic reprogramming by enhancing glucose uptake and glycolytic activity, stimulating the pentose phosphate pathway, and supporting anabolic processes and redox homeostasis. In parallel, NRF1 contributes to mitochondrial biogenesis and the maintenance of OXPHOS, thereby supporting mitochondrial function and metabolic adaptability. The coordinated and context-dependent interplay between NRF1- and NRF2-mediated pathways contributes to the metabolic plasticity that enables cells to adapt to fluctuating energetic and oxidative conditions [45,46].

## 4. Conserved Mitochondrial Survival Programs in Normal and Cancer Cells and Intracellular Pathogens

Hypoxia represents another important deeply conserved adaptive mechanism in which mitochondria play a central role in regulating cell survival, ROS production, and metabolic homeostasis [47,48,49,50]. Under low-oxygen conditions, mitochondria undergo profound functional reorganization, including alterations in electron transport chain activity and mitochondrial ROS production. Changes in mitochondrial bioenergetics can activate mitochondrial-to-nuclear retrograde signaling, involving calcium-dependent pathways and stress-responsive transcriptional programs that promote cellular adaptation and survival [51]. Persistent mitochondrial stress can further reshape cellular metabolism by favoring glycolytic glucose utilization and reducing reliance on oxidative phosphorylation, thereby contributing to the metabolic phenotype associated with cancer and the Warburg effect [52]. Alterations in TCA-cycle metabolism and the accumulation of oncometabolites may additionally reinforce oncogenic signaling and remodel the tumor microenvironment [53].

Mitochondria remain metabolically active during this process, sustaining TCA-cycle activity, biosynthesis, redox balance, and proliferation. Alterations in mitochondrial and TCA-cycle enzymes, including IDH, SDH, and FH, promote the accumulation of oncometabolites such as 2-hydroxyglutarate, succinate, and fumarate. IDH, SDH, and FH represent important metabolic nodes whose dysregulation can alter nutrient utilization, redox homeostasis, and cellular signaling [54]. These metabolites inhibit α-ketoglutarate-dependent dioxygenases, inducing epigenetic reprogramming and pseudohypoxic HIF activation [54,55]. However, increasing evidence indicates that alterations in mitochondrial metabolism serve not only as bioenergetic adaptations but also as signaling mechanisms that reshape epigenetic and transcriptional programs, contributing to mitochondrial metabolic plasticity and the metabolic reprogramming to cancer cells [54].

Interestingly, similar metabolic adaptations are observed in microorganisms, providing an evolutionary parallel to the metabolic plasticity of mitochondria and tumor cells. During a bacterial infection, for example, host-derived itaconate can inhibit SDH activity and reshape microbial metabolism. *Pseudomonas aeruginosa* can exploit host-derived itaconate to reprogram succinate metabolism and promote biofilm formation. This metabolic flexibility illustrates how microorganisms can sense and exploit changes in the host’s metabolic environment to enhance survival and persistence. These conserved adaptive strategies parallel mechanisms retained within mitochondria and potentially reactivated or repurposed in tumor cells, enabling metabolic remodeling, stress resistance, and adaptation to hostile microenvironments [55,56]. Host succinate metabolism can also influence antiviral responses, during influenza A virus infection, succinate promotes succinylation and nuclear retention of viral nucleoprotein, thereby restricting viral replication [57]. In fungi, alterations in SDH-encoding genes, including SdhB, can confer resistance to SDH-targeting fungicides such as penthiopyrad and fluopyram [58,59,60].

Under chronic stimulation, this redox network can become maladaptive, with persistent mitochondrial ROS perturbing electron transport, membrane integrity, and the mitochondrial electrochemical gradient (ΔΨm), thereby compromising oxidative phosphorylation [61,62,63]. Reduced mitochondrial bioenergetic capacity can increase reliance on glycolysis and substrate-level ATP production, providing a metabolic adaptation that supports cell survival under mitochondrial stress [64]. In *Saccharomyces cerevisiae*, mitochondrial dysfunction can also promote nuclear genome instability through defects in iron–sulfur cluster biogenesis, highlighting the intimate connection between mitochondrial function and nuclear genome maintenance [65].

## 5. Mitochondrial Survival Stress Mechanisms, from Apoptosis, to the Hypoxic Adaptation in Cancer and Intracellular Pathogens

More broadly, mitochondrial stress activates retrograde responses that coordinate mitochondrial and nuclear adaptation [66]. In cancer cells, reduction in mtDNA content has been shown to promote aerobic glycolysis and reversible resistance to apoptosis, illustrating how mitochondrial dysfunction can be coupled to a glycolytic survival phenotype [67,68]. Among the most relevant pathways are the signal transducer and activator of transcription (STAT1/STAT3) signaling axis, which regulates inflammation, immune responses, and the balance between survival and apoptosis; the second mitochondria-derived activator of caspases/direct iap-binding protein with low pi (SMAC/DIABLO-IAP) signaling pathway, which links mitochondrial outer membrane permeabilization to caspase-dependent cell death; and the hypoxia-inducible factor 1 (HIF-1) pathway, which integrates oxygen availability with mitochondrial metabolism and glycolytic adaptation. These pathways intersect at any time with programmed cell death protein 1 (PD-1) and its ligand, programmed death-ligand 1 PD-1/PD-L1-mediated immune regulation, linking cellular metabolism and mitochondrial stress to immune surveillance and immunoevasion [69,70,71,72,73]. Mitochondria, intracellular pathogens, and tumor cells engage overlapping signaling networks involving all those pathways, influencing apoptosis, inflammation, metabolism, and immune surveillance [69,70,71,72,73].

Among these pathways, the STAT1–STAT3 axis is particularly relevant to the balance between inflammatory activation, apoptosis, and cellular survival. Under chronic inflammatory conditions, persistent STAT3 activation together with impaired STAT1 signaling can shift the cellular response toward survival, inflammatory persistence, and resistance to apoptosis, accompanied by alterations in redox homeostasis [74,75,76,77]. STAT1 and STAT3 therefore function not simply as independent transcription factors but as components of a dynamic signaling network whose balance can determine whether a stressed cell undergoes elimination or adapts to persistent environmental pressure [77].

In squamous cell carcinoma, STAT1 promoter methylation has been associated with reduced STAT1 expression, providing an epigenetic mechanism through which tumor cells can attenuate STAT1-dependent responses [78]. During *Mycobacterium tuberculosis* infection, STAT1 signaling can also be functionally altered, including impairment of IFN-γ-dependent transcriptional responses and modulation of STAT1-associated apoptotic pathways [79,80]. However, whereas promoter methylation provides direct evidence for epigenetic STAT1 silencing in squamous carcinogenesis, the available evidence for *Mycobacterium tuberculosis* indicates functional interference with STAT1-dependent signaling rather than establishing promoter hypermethylation as the universal mechanism. This distinction illustrates how different biological systems may converge functionally on suppression of STAT1-mediated antimicrobial or pro-apoptotic responses while using different molecular mechanisms [80].

The consequences of altered STAT1/STAT3 signaling extend to immune surveillance. Mitochondrial stress can promote the release or extracellular transfer of mitochondrial components, including mtDNA, which can engage innate immune pathways and influence STING-dependent signaling. In cancer, mitochondrial alterations have been linked to PD-L1-associated immune escape, while extracellular vesicles containing mitochondrial components and PD-L1 can modulate macrophage and T-cell responses [81]. Thus, mitochondrial dysfunction may simultaneously influence intracellular metabolism and extracellular immune communication, creating a cellular environment characterized by persistent inflammatory signaling, immune suppression, and enhanced survival [81].

SMAC/DIABLO represents a second major point of convergence between mitochondrial integrity and apoptotic regulation. SMAC/DIABLO is normally localized in the mitochondrial intermembrane space and is released into the cytosol following mitochondrial outer membrane permeabilization (MOMP). Once released, it antagonizes inhibitor of apoptosis proteins (IAPs), particularly X-linked inhibitor of apoptosis protein (XIAP), thereby relieving IAP-mediated inhibition of caspases and facilitating apoptotic execution [82,83,84,85]. Thus, SMAC/DIABLO is fundamentally pro-apoptotic: its function is to remove an important molecular brake on caspase activation rather than to suppress apoptosis. The functional relationship between mitochondrial permeabilization, SMAC/DIABLO release, XIAP inhibition, and caspase activation provides a direct molecular link between mitochondrial damage and programmed cell death [85].

Cancer cells and intracellular pathogens can interfere with apoptotic execution through mechanisms that preserve mitochondrial integrity, enhance anti-apoptotic Bcl-2-family activity, alter IAP-dependent regulation of caspases, or otherwise disrupt mitochondrial death signaling [86,87,88,89,90]. Such mechanisms may prevent the release or functional activity of mitochondrial apoptogenic proteins and thereby increase cellular resistance to apoptosis. In this context, the biological effect of SMAC/DIABLO cannot be inferred solely from its cellular abundance. Effective apoptotic signaling depends on its mitochondrial localization, release following MOMP, interaction with IAPs, and the functional competence of downstream caspases [82,83,84,85]. Consequently, increased SMAC/DIABLO expression does not necessarily translate into proportional restoration of apoptosis when other components of the mitochondrial apoptotic machinery remain dysfunctional [91,92,93,94].

The HIF-1 pathway represents a third major component of the mitochondrial–nuclear stress network. Hypoxia profoundly alters mitochondrial metabolism, redox balance, and epigenetic regulation, while mitochondrial ROS and metabolic intermediates can influence HIF-1 activation [81,95,96,97]. Conversely, HIF-1 regulates glucose metabolism and multiple aspects of mitochondrial function, establishing a bidirectional signaling relationship between mitochondrial state and nuclear transcription. This relationship allows changes in oxygen availability, mitochondrial activity, and cellular metabolism to be integrated into coordinated transcriptional programs that promote adaptation and survival [81].

The interaction between inflammatory and hypoxic signaling further reinforces this network. NF-κB can promote HIF-1α transcription (a sub-unit of HIF-1), linking innate inflammatory signaling to the cellular response to hypoxia [81,95,96,97]. In cancer, sustained HIF-1 α specifically promotes glycolytic metabolism and adaptation to hypoxic microenvironments, while mitochondrial dysfunction can itself influence the ability of cells to activate HIF-1-dependent transcription [81,95,96,97]. Mitochondrial metabolism and HIF-1 signaling should therefore be considered as components of a reciprocal regulatory system rather than as independent pathways. This relationship provides a mechanistic basis through which mitochondrial stress can influence cellular metabolism, proliferation, angiogenic responses, and resistance to adverse environmental conditions [98].

Actually, HIF-1 signaling also intersects with immune checkpoint regulation, it enhances PD-L1 expression in several tumor and immune-cell contexts, thereby contributing to an immunosuppressive microenvironment and facilitating immune escape from T-cells [98,99]. Consequently, the mitochondrial–HIF-1 axis can connect metabolic adaptation to immune regulation, mitochondrial downregulation and hypoxia can alter HIF-dependent transcription, while HIF-1-driven metabolic and immune programs can reinforce cellular survival within the tumor microenvironment. This reciprocal relationship is particularly relevant in tumors, where hypoxia, inflammation, mitochondrial aberration, and immune suppression coexist chronically [99].

### Mitochondrial Metabolism as a Driver of Epigenetic Reprogramming and Cancer Cell Plasticity

During periods of mitochondrial metabolic perturbation, such as those occurring within the infectious and tumor microenvironment, where cellular stress, hypoxia, and immune-evasion networks intersect, the availability of critical cofactors and metabolites, including acetyl-CoA and NAD^+^, can become altered, directly influencing the balance between histone acetylation and deacetylation [100,101]. Mitochondria-derived acetyl-CoA provides a substrate for histone acetyltransferases and can promote active chromatin marks, including H3K27 acetylation, whereas changes in the NAD^+^/NADH ratio modulate sirtuin-dependent histone deacetylation. This metabolic–epigenetic coupling provides a direct link between cellular energy metabolism and the regulation of gene expression [101]. Through these mechanisms, mitochondrial metabolic status can reshape transcriptional programs governing proliferation, differentiation, and cellular stress adaptation, including the expression of key regulators such as *Nanog*, *Oct4*, and *Runx2* [102,103].

Notably, a critical step linking mitochondrial metabolic alterations to long-term epigenetic remodeling is the acquisition of malignant phenotypes, including anchorage-independent growth (AIG), a key hallmark of cellular transformation. Sustaining proliferation under non-adherent conditions requires increased mitochondrial mass and enhanced H3K27 acetylation, which support the elevated metabolic and biosynthetic demands of transformed cells [103,104,105]. De Luca et al. demonstrated that mitochondria coordinate this adaptive process by coupling energy production to biosynthetic pathways, particularly glutamine metabolism, through mechanisms involving mitochondrial sirtuins and enzymes such as glutamate dehydrogenase [106]. This metabolically driven mitochondrial biogenesis is also critical for the maintenance and propagation of cancer stem-like cells, further linking mitochondrial plasticity to tumor initiation, adaptation, and progression [107].

## 6. mtDNA Transfer into the Nucleus, NUMT Formation, Genomic Instability, and Their Potential Contribution to Carcinogenesis

During chronic infection, persistent mitochondrial stress may progressively alter the capacity of the nucleus to maintain normal genomic and transcriptional homeostasis. Rather than representing a passive consequence of cellular damage, mitochondria may increasingly influence nuclear responses through retrograde signaling, metabolic regulation, and the transfer of mitochondrial genetic material. In this context, an intriguing question arises: how might mitochondria progressively reshape nuclear function? As proposed by Walker and colleagues, one possible mechanism involves the integration of mitochondrial genetic material into the nuclear genome, generating nuclear mitochondrial DNA segments (NUMTs) [108,109,110,111]. While the physical translocation of NUMTs start with their accumulation around the nuclear envelope during bioenergetic stress, and their subsequent integration into nuclear double-strand breaks are well-documented genomic phenomena, we hypothesize that this pathway acts as a recurring, non-random driver of malignant transformation under chronic metabolic stress.

In fact, the accumulation of NUMTs may represent a molecular footprint of continued mitochondrial stress and a potential mechanism through which mitochondrial genetic information becomes permanently incorporated into the nuclear genome. Although NUMTs acquire the inheritance pattern of the nuclear genome after integration, their origin reflects a form of intracellular lateral genetic transfer, reminiscent of the genome plasticity observed in bacteria [112]. These mtDNA-derived insertions establish a permanent genetic interface between mitochondria and the nucleus and may influence gene regulation, genomic stability, and cellular adaptation. Once integrated, NUMTs can persist within the nuclear genome, undergo duplication, accumulate mutations, and occasionally acquire new regulatory or functional properties [113,114,115]. This process highlights a striking evolutionary continuity between prokaryotic genome plasticity and eukaryotic mitochondrial–nuclear interactions, suggesting that the transfer and remodeling of mitochondrial genetic information may contribute to cellular adaptation under persistent environmental and metabolic stress [113,114,115]. Nearly a century ago, scientists identified NUMTs in murine lymph node cells, and later in ascitic and solid tumors of the tongue, pancreas, and liver during tissue regeneration [113,114].

The presence of NUMTs confirmed that mtDNA can be transferred to the nucleus, although the precise mechanisms underlying this process remain incompletely understood. These sequences are thought to arise either through reverse transcription of mitochondrial RNA transcripts followed by cDNA integration and editing, or through the direct incorporation of double-stranded mtDNA fragments into the nuclear genome via non-homologous end joining (NHEJ) at sites of double-strand DNA breaks [115,116].

Researchers amplified several NUMTs from individuals of diverse ethnic backgrounds, including Caucasian, African, and Asian populations, and confirmed remarkably consistent NUMT lengths across nearly all samples [117]. Later studies identified mitochondrial sequences within the nuclear genomes of blood lymphocytes, Hodgkin’s lymphoma cells, leukemic myoblasts, and myocarditis tissues [117,118]. Moreover, mtDNA, mtRNA, and mitochondrial proteins have been detected within the nuclear compartments of monocytes, particularly under conditions of cellular stress, disease, or specific signaling activation. These observations suggest that mitochondrial components may directly participate in nuclear regulatory processes, including epigenetic remodeling, modulation of gene expression, activation of innate immune pathways, and cellular stress adaptation [117,118].

**Figure 2:**
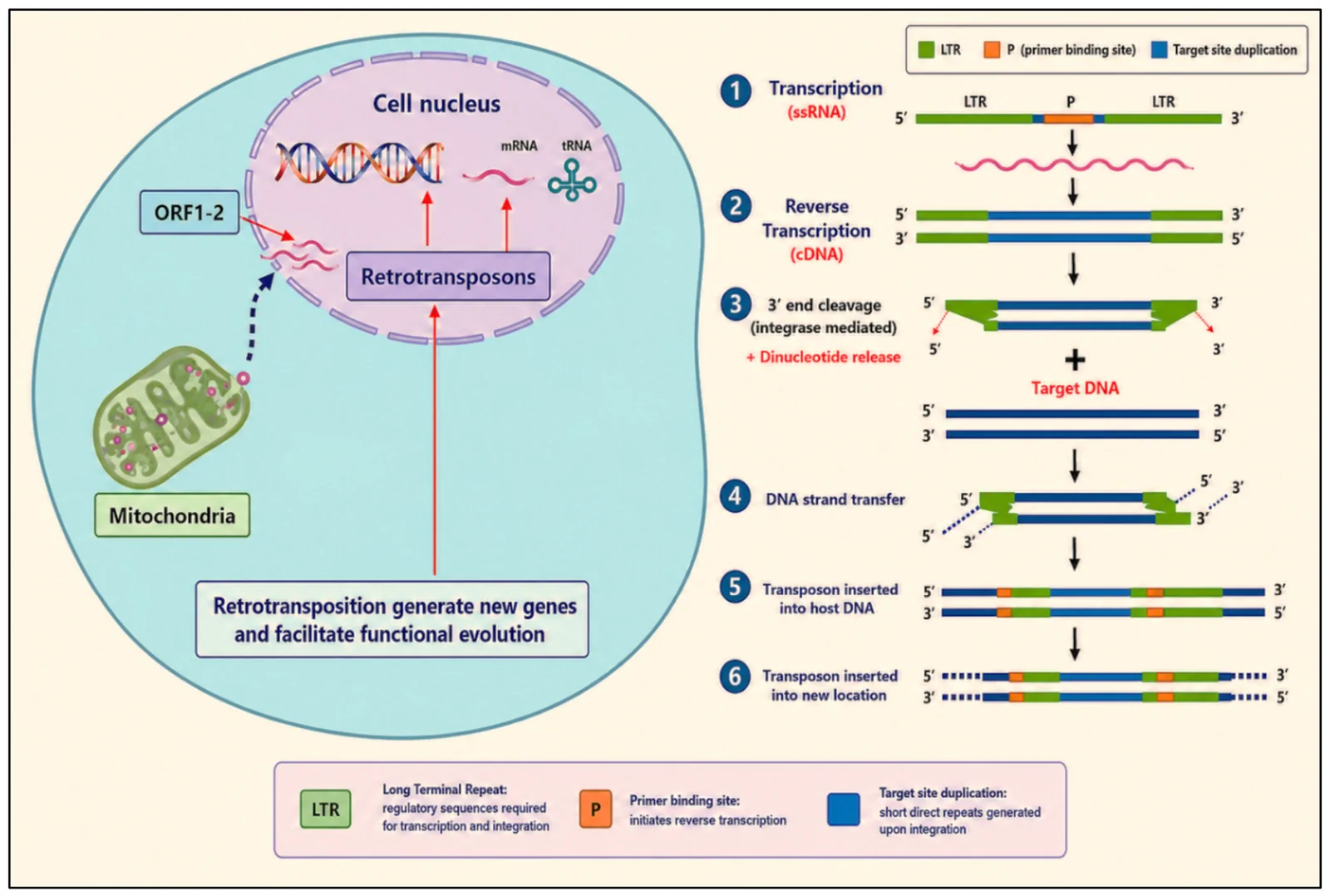
Proposed model of NUMT integration into the nuclear genome through a mechanism analogous to retrotransposon-mediated insertion. The process may occur through six sequential steps: (1) transcription of mitochondrial-derived RNA intermediates; (2) reverse transcription into complementary DNA (cDNA); (3) cleavage of nuclear target DNA, potentially mediated by retrotransposition-associated endonuclease activity; (4) integration of mitochondrial-derived cDNA into the nuclear genome; (5) gap filling and repair of unpaired DNA regions; and (6) final ligation and stabilization of the mitochondrial insert within its new nuclear locus.

The preferential accumulation of NUMTs within pericentromeric and subtelomeric regions suggests their integration into both coding and non-coding nuclear sequences. Although integration into coding regions, including introns or exons, appears to be a relatively rare event, such insertions may nevertheless lead to two major outcomes: (i) the incorporation of mitochondrial fragments as novel exons within existing genes, and (ii) a form of “nuclear hijacking,” through which mitochondrial-derived sequences may modulate nuclear transcriptional activity, influence proto-oncogene activation, and reshape nuclear regulatory networks [118]. A shorter repeat length and a reduced copy number of heteroplasmic mtDNA improve mitochondrial efficiency and resilience under restrictive conditions of hypoxia, the effects of chemotherapy, or other adverse epigenetic conditions [115,118] (Figure 2).

### Transposable Elements and NUMTs in Eukaryotic Cells

To further confirm this hypothesis, another important factor should be considered, the transposable elements (TEs). Together with NUMTs, TEs are major contributors to genome evolution and structural remodeling because they can move and rearrange DNA sequences. The connection between TEs and NUMTs further highlights the coevolutionary relationship between microorganisms and eukaryotic cells, suggesting that ancient endosymbionts and mobile genetic elements have followed moderately overlapping evolutionary pathways within the host genome [119].

TE-rich genomic regions may favor the fragmentation, duplication, and further remodeling of NUMTs after their integration into the nuclear genome [119]. In turn, NUMTs located within these regions may become subject to recombination and epigenetic changes, influencing their stability and function. Recent findings have shown that TEs have role in having shaped the architecture and function of eukaryotic genomes, acting simultaneously as agents of mutation, generators of genetic diversity, and regulators of gene expression. Thus, the interaction between NUMTs and TEs may represent an additional mechanism through which mitochondrial genetic material is progressively reshaped within the nuclear genome, contributing to genome plasticity and cellular adaptation to persistent metabolic stress [120,121].

The human genome predominantly contains two major classes of RNA-mediated TEs, long interspersed nuclear elements (LINE-1 or L1) and Alu elements, which belong to the short interspersed nuclear element (SINE) family [121]. LINE-1 elements are autonomous retrotransposons capable of copying and inserting themselves into new genomic locations through their own enzymatic machinery, accounting for approximately 16–17% of the human genome. Alu elements, by contrast, are non-autonomous SINEs unique to primates and represent the most abundant mobile genetic elements, comprising roughly 9–11% of the genome [121,122]. Collectively, TEs constitute nearly 50% of primate genomes, whereas protein-coding sequences represent only ~2% [123,124].

Therefore, TE activity has been considered a major driver of genome evolution, facilitating the emergence of new genes and regulatory networks. In eukaryotes, TEs modulate gene expression and chromatin dynamics, influencing cellular plasticity and adaptability [125]. In microorganisms TE insertions sequences (IS) frequently target central metabolic and informational hubs, producing mutagenic or deleterious effects, especially considering the initial impact of TEs on microbial viability often producing harmful effects on the host [125].

In primates, L1 activity has been implicated in gene duplication events that enhance genomic diversity and adaptive potential. Comparable processes occur in bacterial genomes, where insertional mutagenesis by TEs can disrupt essential genes and initiate pro-inflammatory signaling pathways that interfere with mitochondrial function [126,127]. Thus, despite their parasitic reputation, TEs represent a fundamental evolutionary force shaping the structure, function, and adaptability of the human genome and karyotype [126,127].

TEs can be broadly divided into two major classes, DNA transposons and retrotransposons, and based on their mechanism of genomic integration [128]. The molecular and evolutionary characteristics of transposons and retrotransposons in mammals play crucial roles in the emergence of new silent genes and retrogenes [128,129]. Because of their mobility, TEs can influence nearby regulatory sequences, leading to transcriptional dysregulation, protein misfolding, and so contributing to the development of spontaneous genetic disorders and cancer. Approximately 45% of the human genome is derived from TEs, with L1 elements representing the only currently active autonomous retrotransposons in humans [130]. L1 elements belong to the non-LTR (non-long terminal repeats) retrotransposon (jumping genes) class and are responsible for the mobilization of many retrocopies and processed pseudogenes within the genome [130,131]. In contrast, long terminal repeats (LTR) retrotransposons can generate retrocopies that are often co-transcribed with adjacent LTR sequences [130,131]. In response to retrotransposon activation, cancer cells activate multiple epigenetic silencing mechanisms, including DNA methylation and histone modifications, to repress transposon expression [131].

Notably, mitochondria appear to play a central role in this entire process. Recent findings strongly suggested that mitochondrial function can modulate TE activity bidirectionally, either suppressing or enhancing transposition depending on cellular conditions [131,132]. Under conditions of mitochondrial stress, cytosolic signaling pathways are rapidly activated, leading to alterations in nuclear gene expression that frequently involve TE regulation [132]. This stress-induced feedback may paradoxically promote genomic instability by increasing cellular susceptibility to additional perturbations. Such mechanisms are closely associated with mitochondrial signaling mediators, including Smac/DIABLO, HIF-1, and STAT1, which participate in the regulation of apoptosis, hypoxic responses, and inflammatory pathways through complex transcriptional and signaling networks [132,133,134] (Table 1).

## 7. Proposed Phased Model of Mitochondrial Somatic Mutation Leading to the Formation of Dedifferentiated Cancer Supercells

Within the present interpretative model, there is a progressive mitochondria-centered reprogramming process in which cellular survival is prioritized over multicellular functional integration. Such reprogramming is accompanied by gradual mitochondria dedifferentiation, disruption of tissue-specific transcriptional networks, and destabilization of species-order regulatory systems governing cellular identity and specialization [135,136]. The resulting loss of coordinated nuclear cellular behavior and biological *telos* (τέλος; completion, end, or functional purpose) may therefore represent more than a secondary consequence of oncogenic transformation, instead reflecting a profound collapse of evolutionarily conserved mechanisms responsible for maintaining species-specific multicellular organization [137,138].

As Casás-Selves and Degregori prosed, cancer evolution is driven not only by the accumulation of oncogenic mutations but also by interactive alterations due to selective pressures within the tissue microenvironment [138]. Microorganisms exhibit analogous adaptive strategies, having evolved under persistent selective pressures that drive the continual emergence of multidrug-resistant phenotypes.

In prokaryotes, transposons often harbor genes encoding antibiotic resistance proteins, and these mobile elements may integrate into plasmids to facilitate horizontal gene transfer between host cells without compromising long-term species stability and genetic continuity [139,140]. Consistent with this paradigm, cancer cells, similarly to pathogenic microorganisms, can acquire resistance to therapeutic interventions, including chemotherapy and immune checkpoint blockade, through mechanisms involving extracellular vesicles and exosomes, which mediate intercellular transfer of drug-resistance information, oncogenic signals, and immunomodulatory molecules [141].

These intrinsic mutations are mediated through signaling cascades that engage downstream effector proteins and involve profound isotopic switching triggered by different death stimuli, including dioxygen exposure, metabolic perturbations, deprivation of survival factors, oxidative stress, Ca^2+^ and Mg^2+^ overload, DNA damage, proteotoxic stress, and oncogene activation [142,143]. Alhatem and colleagues have proposed that when cells experience prolonged severe energetic stress, mitochondria may activate, through evolutionary ancient survival programs inherited from unicellular ancestors, the response enabling the host cell to mount adaptive responses aimed at resisting and ultimately overcoming the invading stressor [144].

Therefore, intracellular pathogens may act as chronic ecological stressors that continuously drive remodeling of the cellular and tissue microenvironment in ways that initially favor their own persistence. This inflammatory first phase may persist for years and, in most cases, does not culminate in tumor development [144]. Under conditions of chronic inflammation, the second phase is characterized by progressive mitochondrial and metabolic adaptation, which reshapes the host response and selects cellular populations increasingly resilient to the persistent external insult. Sustained tissue injury, compensatory repair, and the progressive stabilization of these adaptive phenotypes may ultimately contribute to malignant transformation as a maladaptive defensive response [144,145,146,147]. As tumor progression advances, the third phase is characterized by progressive microenvironmental reorganization, which may become increasingly unfavorable to the original infectious agent while remaining highly supportive of malignant cell survival and expansion [148,149]. Once malignant transformation becomes established, the original pathogen may no longer be detectable at the site of the developing tumor, as observed in several infection-associated malignancies involving the stomach, cervix, oropharyngeal tissues, and other anatomical sites. This phenomenon may be conceptualized as a “hostile takeover,” in which a microenvironment initially remodeled by chronic infection becomes progressively reorganized and sustained by the emerging neoplastic cell population, which ultimately displaces the original external intruder [149,150]. Thus, the pathogen may function as an initiating ecological pressure whose presence is no longer required once the tumor has established an autonomous and self-sustaining microenvironment (Table 2).

### 7.1. From Stress Adaptation to Multipotent Cancer Stem Cell Formation, the Double Life of Mitochondria

**Figure 3:**
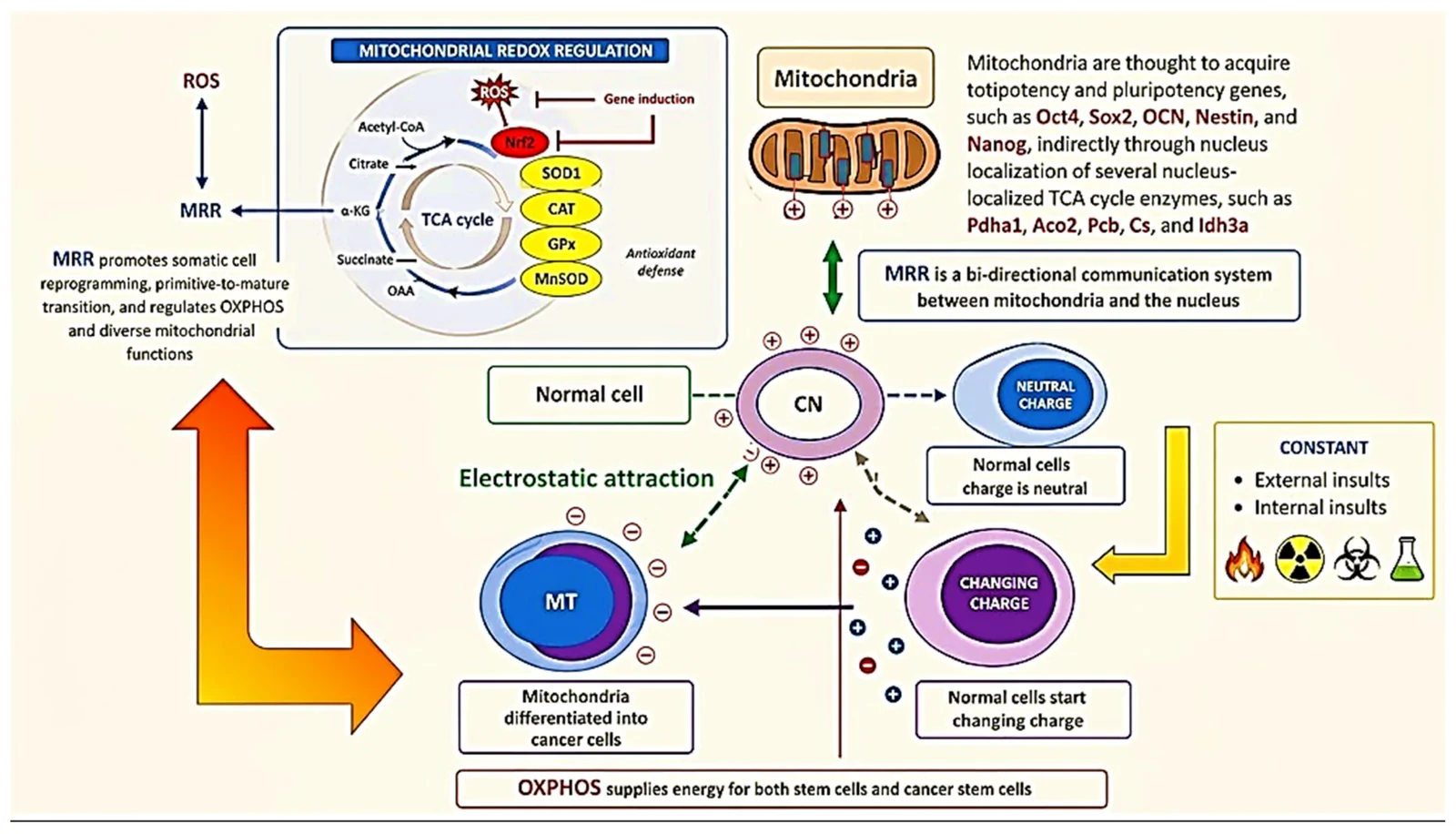
Multiple interconnected mechanisms regulate the process of mitochondrial differentiation toward tumor-like phenotypes. These mechanisms may represent components of the primordial mitochondrial arsenal inherited from its ancestral bacterial–archaeal origin, including adaptive systems involved in stress response, metabolic reprogramming, genomic plasticity, redox regulation, and survival signaling. In this model, mitochondria respond dynamically to persistent cellular stress, nuclear dysfunction, hypoxia, oxidative imbalance, and chronic inflammatory insults, promoting phenotypic adaptation and cellular survival. Such processes may contribute to somatic reprogramming, altered electrostatic and metabolic states, OXPHOS modulation, stemness acquisition, and progressive transition toward cancer cell formation.

The metabolic shift from OXPHOS to SLP and glycolysis represents a crucial prerequisite for the efficient reprogramming of somatic cells into induced pluripotent stem cells (iPSCs) [151]. Presumably, the dedifferentiation process through which cells reacquire stem cell–like features following mitochondrial manipulation may involve the reactivation of genetic programs associated with multipotency, pluripotency, and totipotency [152]. Although direct experimental evidence remains limited, several investigators have proposed that mitochondria may indirectly regulate the expression of stemness-associated transcription factors, including *Oct4*, *Sox2*, *Nanog*, *Klf4*, and *c-Myc*, through the nuclear translocation of TCA cycle enzymes such as PDHA1, ACO2, PCB, CS, and IDH3A [151,152] (Figure 3).

The nuclear relocalization of these metabolic components appears capable of promoting epigenetic remodeling via the alterations in acetylation, methylation, and chromatin accessibility, facilitating the acquisition of stem cell–like properties characterized by self-renewal capacity and broad differentiation potential [153]. The gain of stemness-associated functions may partially explain the remarkable invasiveness, metabolic plasticity, and therapeutic resistance observed in cancer stem cell populations under mitochondrial influence [153]. This interpretation is consistent with the mitochondrial–stem cell connection theory proposed by Martinez and colleagues, according to which chronic OXPHOS insufficiency in stem cells promotes adaptive metabolic reprogramming that may ultimately culminate in the emergence of cancer stem cell–like populations characterized by an aberrant energetic state [148] (Figure 2).

The foundational premise of the proposed hypothesis lies in distinguishing between (i) functional death, a programmed form of cellular elimination in which nuclear and mitochondrial disassembly occur in a coordinated manner, thereby preserving tissue integrity and organismal homeostasis. While apoptosis represents the physical and irreversible dismantling of the cell, functional death denotes the complete loss of tissue-specific physiological differentiation and homeostatic contribution while the cell remains metabolically active; and (ii) non-functional survival, a maladaptive state in which chronic mitochondrial dysfunction disrupts mitochondria–nuclear communication, driving progressive nuclear reprogramming toward stress-adaptive programs that may ultimately culminate in a dedifferentiated, multipotent or pluripotent stem cell-like phenotype. Therefore, non-functional survival describes an uncoupled, stress-driven state where the cell abandons differentiated functions exclusively in favor of primitive, self-preservation bioenergetics, rendering apoptosis resistance and stress adaptation downstream outcomes of this fundamental phenotypic shift [154].

Within this perspective, apoptosis may be interpreted as a mitochondria-regulated cathartic process (κάθαρσις) essential for maintaining multicellular biological order, that permit the persistence of metabolically compromised cells that progressively escape differentiated tissue-specific orders. In support of this interpretation, Rahman and Quadrilatero, although in a different biological context, proposed that mitochondria undergo profound structural, metabolic, and functional remodeling during transitional cellular states characterized by extensive bioenergetic reorganization [155]. During these transitional phases, mitochondria progressively shift from a tightly regulated endosymbiotic integration within the eukaryotic cell toward a state of functional decoupling, characterized by altered bioenergetic autonomy, disrupted mito-nuclear communication, and a progressive loss of coordinated cellular homeostasis [156].

When nuclear regulatory control declines, the resulting transitional state may redirect phenotypic trajectories away from apoptosis or senescence toward uncontrolled proliferation, via the modification exerted on telomere and telomerase mechanisms, reflecting a progressive functional decoupling of mitochondrial activity from nuclear governance [157]. Mitochondrial autonomy, or the ability to function independently of nuclear control, should therefore be interpreted within the view of mitochondrial pangenesis, defined not by a telos-oriented behavior, but rather by a persistent potential for structural and functional self-remodeling. In this context, mitochondrial autonomy may represent an emergent consequence of evolutionary history and microbial species-specific constraints, rather than a redefined cellular “fate” [158,159].

According to Erenpreisa and Cragg, rather than undergoing irreversible degeneration and death, a subset of senescent cancer cells may enter a state of polyploidization, giving rise to giant polyploid cells containing multiple genome copies [160]. This polyploid state may represent a persistent survival and cellular reprogramming phase that enables subsequent recovery of non-stop proliferative potential and stem cell–like properties [160]. Consistent with this idea, Marques and colleagues evidenced that inherited mtDNA mutations can extensively remodel inflammatory cytokine responses and immunometabolic programming [147]. In neoplastic contexts, exosomes, similarly to bacterial plasmids in microbial systems, function as central mediators of tumor growth and homeostasis by coordinating intercellular communication networks that favor cellular survival and adaptation [147]. Through the horizontal transfer of oncogenic proteins, nucleic acids, mitochondrial components, and immunomodulatory signals, tumor-derived exosomes orchestrate metastatic dissemination while simultaneously modulating immune responses, promoting epithelial–mesenchymal transition (EMT), sustaining angiogenesis, and conferring chemoresistance [145].

### 7.2. Bidirectional Signaling Across the Mitochondria–Telomere Axis: Roles of TIN2, TPP1, and TERT in Cellular Longevity

The long and highly dynamic process leading to cancer stem-cell formation involves mechanisms closely linked to the regulation of cellular lifespan, particularly the telomere–telomerase system, which plays a central role in maintaining telomere integrity, cellular longevity, and proliferative potential. Mitochondria interact functionally with telomeres and telomerase through a bidirectional signaling network, linking mitochondrial metabolic and stress status to telomere maintenance, senescence, and replicative capacity [161]. Chen and colleagues identified a protein TIN2 as a key component linking telomere maintenance to mitochondrial metabolic regulation and needs, with implications for cellular lifecycle regulation and replicative potential [161]. Though mitochondrial dysfunction and excessive ROS production can promote telomere damage and accelerate telomere shortening, HIF-1α (subset of HIF-1) binds to telomerase components, including telomerase reverse transcriptase (TERT), can translocate to mitochondria and modulate mitochondrial function and oxidative stress prolonging cells life cycle [162,163].

However, the crucial player in this mitochondria-TERTs mechanism are two main components the TIN2 (TRF1-interacting nuclear protein 2) localized in the mitochondria, and TPP1 (TIN2- and POT1-interacting protein) localized in the lysosomes, both are part of the shelterin system, and protect chromosome ends and regulate telomerase access to telomeres [161,164]. While telomere shortening is suggestive of DNA damage and mitochondrial dysfunction, telomere elongation engages telomeric proteins, particularly TIN2, that are also localized in mitochondria, where they regulate metabolism and the production of ROS [161,164].

TIN2 and TPP1 levels are critical for the efficient reprogramming and long-term maintenance of pluripotent stem cells, as demonstrated in iPSC models. Studies have shown that they preserve telomere integrity and supports the replicative capacity required for sustained pluripotency [165,166]. In addition to its canonical role within the shelterin complex, TIN2 exerts extratelomeric functions, including mitochondrial localization and regulation of cellular energy metabolism, suggesting that it may influence stem cell fate not only through telomere protection and genomic stability but also by modulating the metabolic adaptations required to maintain stemness [165,166]. Importantly, mitochondrial dysfunction may act upstream of telomere regulatory pathways, altering the expression or function of TPP1. Because TPP1 is essential for the recruitment and regulation of telomerase at telomeres, disruption of the TPP1–TIN2 axis may compromise telomere maintenance and thus influence replicative potential and cellular reprogramming [165,166].

Note, cancer stem cells reactivate or upregulate telomerase/TERT expression to counteract end-replication loss. In this context, mitochondrial dysfunction may drive deviating cellular outcomes, ranging from age-associated phenotypes to the emergence of immortalized cancer stem cell states, although the precise mechanisms still remain incompletely understood [167,168]. Similarly to the positions proposed by Björn Brücher and Ijaz Jamall, this current hypothesis implies a paradigm shift with major clinical and epistemological implications, in which cancer may be reinterpreted as a mitochondria-driven mode of persistence, analogous to microorganisms that fulfill survival imperatives genetically linked to the principle of species continuity [169]. Unlike microorganisms, whose persistence ultimately supports species continuity through replication, adaptation, and lineage expansion, mitochondrial/cancer persistence may instead reflect a strategy oriented toward maintenance of a cellular “status quo” [170].

These dedifferentiated cancer stem cells develop the capacity to deviate from the higher-order of human evolutionary objectives governing multicellular organismal integrity. The persistence of metabolically reprogrammed cancer cells may represent the progressive emergence of a quasi-autonomous survival state in which ancestral mitochondria-driven adaptive programs replace the cooperative regulatory constraints required for differentiated multicellular homeostasis [170,171,172].

The molecular parallels between mitochondria, tumor cells and intracellular pathogens become particularly relevant, since all can manipulate metabolism, mitochondrial signaling, apoptosis, and host immune responses to prolong survival. These similarities may represent convergent exploitation of conserved cellular survival mechanisms and provide an evolutionary framework in which mitochondrial reprogramming constitutes a central interface between metabolic adaptation, immune evasion, and malignant progression [170,171,172]. Such a transition may ultimately favor cellular perseverance at the expense of tissue specialization, organismal coordination, and species-level biological functionality. As proposed by Da Silva, cancer does not merely represent uncontrolled cellular proliferation, rather the expression of a primordial bioenergetic survival program centered on mother mitochondrial autonomy [170,171,172,173,174]. Therefore, in line with the evolutionary interpretation of cancer proposed by Wang and colleagues and Ishida and colleagues, mitochondrial legacy may promote a reversion toward evolutionarily ancient, unicellular-like cellular programs characterized by metabolic flexibility, enhanced glycolytic activity, altered TCA-cycle flux, resistance to apoptosis, and increased biosynthetic capacity, thereby creating a cellular state that favors malignant transformation [175,176].

## 8. Conclusions

Cancer development has traditionally been interpreted through the lens of nuclear genetic alterations. However, growing evidence from cancer immunobiology, mitochondrial research, immunometabolism, and host–microbiota interactions suggest that many hallmarks of malignancy may also represent components of a broader adaptive survival response centered on mitochondrial stress signaling and metabolic reprogramming. Within this position, mitochondria are viewed not as passive organelles damaged during oncogenesis, but as dynamic regulators of cellular adaptation capable of reshaping redox balance, immune signaling, apoptosis, and transcriptional programs under chronic stress conditions. On this basis, we propose that carcinogenesis may arise through a long-term progressive and multifactorial process involving, (1) persistent environmental, infectious, inflammatory, or metabolic stressors; (2) chronic disruption of mitochondria–nucleus communication accompanied by progressive mitochondrial remodeling linked to their endosymbiotic origin; (3) activation of adaptive stress-survival pathways associated with metabolic plasticity and apoptosis resistance, resembling mechanisms commonly observed in intracellular microorganisms; (4) establishment of chronic adaptive programs that promote immune evasion, tissue disorganization, and cellular persistence; and ultimately (5) a mitochondria-centered dedifferentiation process characterized by partial loss of specialized cellular identity and the rise in malignant phenotype. In this model, nuclear genomic instability remains fundamentally important but may represent both a trigger and a downstream consequence of prolonged adaptation to mitochondrial stress. Cancer therefore may not solely reflect uncontrolled proliferation driven by stochastic mutations, but also a deeply conserved biological response in which ancient mitochondrial survival mechanisms progressively supersede the cooperative constraints required for differentiated multicellular homeostasis. If experimentally validated, this integrative framework could broaden current interpretations of tumorigenesis and identify multiple stages at which carcinogenic progression may be prevented, modulated, or therapeutically reversed. We know that, to validate mitochondrial dysfunction as an upstream driver rather than a secondary consequence, three falsifiable conditions must be met: (i) primary mitochondrial stress (mtROS/succinate accumulation) directly precedes L1 retrotransposon reactivation and nuclear instability in non-transformed cells; (ii) blocking mtROS or L1 reverse transcriptase prevents nuclear transformation; and (iii) cybrids with damaged mitochondria display L1-driven nuclear instability without prior nuclear mutations. If mitochondrial signaling requires pre-existing nuclear mutations to drive transformation, it must be reclassified as a secondary adaptation.

## Figures and Tables

**Table 1:** Key references linking STAT1, HIF-1, and SMAC/DIABLO to mitochondrial signaling, apoptosis, and pathogen activity.

Molecule/Focus	Summary of Findings	Representative Pathogens
STAT1 and *Mycobacteria*	*Mycobacterium tuberculosis* suppresses IFN-γ–responsive gene transcription despite intact STAT1 phosphorylation and nuclear translocation.	*Mycobacterium tuberculosis*
STAT1 and Apoptosis in Tuberculosis	Unphosphorylated STAT1 accumulates during infection, correlating with reduced macrophage apoptosis and enhanced pathogen persistence.	*Mycobacterium tuberculosis*
HIF-1 and Cellular Adaptation to Hypoxia	Pathogens and tumor cells stabilize HIF-1α under hypoxic or inflammatory conditions, promoting glycolytic metabolism, angiogenesis, and immune evasion.	*Mycobacterium tuberculosis*, *Helicobacter pylori*, *Epstein–Barr virus* (EBV), *Listeria monocytogenes*
SMAC/DIABLO Release and Mitochondrial Apoptosis	SMAC/DIABLO release from mitochondria is caspase-dependent and inhibited by anti-apoptotic Bcl-2 proteins; this mechanism modulates apoptosis in viral and bacterial infections.	Viral: HBV, HCV, HPV; Bacterial: various species
SMAC/DIABLO in Cancer Progression	SMAC/DIABLO regulates apoptosis and contributes to tumorigenesis by inhibiting IAPs and facilitating caspase activation.	*Human papillomavirus* (HPV), *Epstein–Barr virus* (EBV), *Helicobacter pylori*

**Table 2:** Multiphase Progression of Infection-Associated Tumorigenesis and Microenvironmental Reorganization.

Phases	Environmental and Cellular Trigger	Biological and Metabolic Process	Outcome and Microenvironmental Impact
Phase 1: Chronic Infection and Inflammation	Persistent pathogen infection (e.g., *H. pylori*) and continuous external insult.	Initiation of sustained tissue injury, recruitment of immune cells, and generation of chronic inflammatory cascades.	Remodeling of the local tissue niche; creation of a high-stress, inflammatory microenvironment.
Phase 2: Progressive Adaptation and Selection	Ongoing inflammation and sustained tissue damage.	Mitochondrial structural/metabolic remodeling; selection of host cell populations resilient to persistent stress; maladaptive repair responses.	Stabilization of stress-resistant phenotypes; initiation of malignant transformation as a defensive survival mechanism.
Phase 3: “Hostile Takeover” & Reorganization	Established neoplastic cell expansion and autonomous tumor signaling.	Complete reorganization of the tumor microenvironment (TME) driven by transformed cells; shift to self-sustaining metabolic programs.	The TME becomes hostile to the original pathogen, leading to its clearance/displacement; the tumor becomes fully autonomous and self-sustaining.

## Data Availability

Data can be asked to the authors if needed.
